# Carbonic Anhydrase-IX Guided Albumin Nanoparticles for Hypoxia-mediated Triple-Negative Breast Cancer Cell Killing and Imaging of Patient-derived Tumor

**DOI:** 10.3390/molecules25102362

**Published:** 2020-05-19

**Authors:** Katyayani Tatiparti, Mohd Ahmar Rauf, Samaresh Sau, Arun K. Iyer

**Affiliations:** 1 Department of Pharmaceutical Sciences, Use-inspired Biomaterials & Integrated Nano Delivery (U-BiND) Systems Laboratory, Eugene Applebaum College of Pharmacy and Health Sciences, Wayne State University, Detroit, MI 48201, USA; katyayani.tatiparti@wayne.edu (K.T.); ahmarrauf2@gmail.com (M.A.R.); samaresh.sau@wayne.edu (S.S.); 2 Molecular Therapeutics Program, Barbara Ann Karmanos Cancer Institute, Wayne State University School of Medicine, Detroit, MI 48201, USA

**Keywords:** TNBC, tumor hypoxia, carbonic anhydrase-IX

## Abstract

Triple-Negative Breast Cancer (TNBC) is considered as the most onerous cancer subtype, lacking the estrogen, progesterone, and HER2 receptors. Evaluating new markers is an unmet need for improving targeted therapy against TNBC. TNBC depends on several factors, including hypoxia development, which contributes to therapy resistance, immune evasion, and tumor stroma formation. In this study, we studied the curcumin analogue (3,4-Difluorobenzylidene Curcumin; CDF) encapsulated bovine serum albumin (BSA) nanoparticle for tumor targeting. For tumor targeting, we conjugated Acetazolamide (ATZ) with CDF and encapsulated it in the BSA to form a nanoparticle (namely BSA-CDF-ATZ). The in vitro cytotoxicity study suggested that BSA-CDF-ATZ is more efficient when compared to free CDF. The BSA-CDF-ATZ nanoparticles showed significantly higher cell killing in hypoxic conditions compared to normoxic conditions, suggesting better internalization of the nanoparticles into cancer cells under hypoxia. Fluorescent-dye labeled BSA-CDF-ATZ revealed higher cell uptake of the nanoparticle compared to free dye indicative of better delivery, substantiated by a high rate of apoptosis-mediated cell death compared to free CDF. The significantly higher tumor accumulation and low liver and spleen uptake in TNBC patient-derived tumor xenograft models confirm the significant potential of BSA-CDF-ATZ for targeted TNBC imaging and therapy.

## 1. Introduction

Cancer still remains one of the major causes of death in the United States, taking more than half a million lives annually, and with approximately 1.5 million new cases diagnosed each year [1]. Several therapeutic and diagnostic arsenals have been developed to fight against cancer. Among different cancer types, the therapeutic success of TNBC is poor [2]. The new therapeutics, including chemotherapy, immunotherapy, and kinase inhibitors, have been developed, but their effect is still merely marginal. The DNA intercalating taxanes, platinum compounds, and tyrosine and serine-threonine kinase inhibitors, such as Everolimus and Cetuximab, are the most common and conventional therapeutic strategies for TNBC. However, in clinical trials, it may lead to drug resistance in TNBC patients. Thus, evaluating new targeting agents and exploiting them for early diagnosis and tumor-specific therapy is an urgent need. Nanomedicines have proven to be a multitasking drug delivery vehicle for tumor therapy. The nanoparticles can passively accumulate in tumor tissues by the well-established Enhanced Permeability and Retention (EPR) effect [3,4,5,6,7,8]. In addition, nanoparticles can be tailored with tumor antigen-specific targeting ligands for active targeting that can improve the tumor selective delivery and therapeutic response [9,10,11,12,13,14,15].

### 1.1. Albumin

Albumin is one of the abundant plasma proteins in human body. It has several anti-immunogenic properties, functions as a transporter of nutrients to various organs of the body and has a good half-life of 19 days. Like most of the plasma proteins, by far, albumin is synthesized in the liver, where it is made at a rate of around 0.7 mg/h for every gram of liver (i.e., 10–15 g step by step). Tumor cells utilize albumin as a rich source of nutrients. Thus, delivering chemotherapeutic drugs loaded in albumin nanoparticle functions as a Trojan horse for killing tumor cells and the FDA approved AbraxaneTM is an outcome of this concept [16,17,18,19]. Besides the clinical success, several challenges, such as (i) undesirable precipitation and instability during storage, (ii) immunogenicity, (iii) high liver uptake, (iv) non-specific binding with other plasma protein, limit the effect of AbraxaneTM [20,21,22]. To solve these challenges, we have decorated ATZ (which is a small molecule inhibitor) on the surface of BSA nanoparticles. The tumor imaging with near-infrared (NIR) dye (S0456) conjugated BSA-CDF-ATZ (namely BSA-CDF-ATZ-S0456) showed superior tumor uptake with a minimal accumulation of NIR dye into the liver and spleen [23,24]. These data support the hypothesis that the hypoxia targeting BSA nanoparticle is an effective approach for developing a protein-based drug delivery system.

### 1.2. Click Chemistry

Our study described in this paper is lined on the concept of self-assembling BSA nanoparticle encapsulated with CDF that is functionalized with targeting ligands utilizing strain-promoted copper-free click chemistry [25,26], specifically the 1, 3 dipolar azide-alkyne cycloaddition. Copper-free click chemistry has several advantages, such as (i) environmental safety, (ii) site-specific conjugation, (iii) high yields, and stereospecificity as compared to other chemical conversions (e.g., maleimide-thiol coupling). The click reaction can be performed in aqueous conditions; thus, it is beneficial for nanomedicine and drug delivery system designing. Considering these advantages, we utilize this strategy to conjugate dibenzocyclooctyne (DBCO) to functionalized Acetazolamide (ATZ) to give ATZ-DBCO that is, in turn, appended to BSA-N3 encapsulated with CDF nanoparticles to target tumor hypoxia.

### 1.3. 3,4-Difluorobenzylidene Curcumin

Curcumin (CMN) has shown an anticancer effect on various cancers. However, poor water solubility, low bioavailability, fast metabolism, and elimination limits the clinical translation [27,28,29]. Due to these limitations, several groups of researchers have attempted to design and formulate CMN analogs possessing better stability and higher bioavailability with biological activity similar to or superior to that of the parent compound [30,31]. Based on these facts, we have designed a novel derivative of 3,4-Difluorobenzylidene Curcumin (CDF) that shows enhanced stability, superior therapeutic potential, and 16-times longer half-life in comparison to CMN. CDF likewise showed highly enhanced tumor-specific accumulation [32,33]. It is a noteworthy finding that tumor development restraint of UM-SCC-1R cells and a decrease in the expression of CD44 were seen in a nude mice xenograft study, showing the promising inhibitory impact of CDF on Cancer Stem Cells (CSCs) [34]. We further found that CDF could hinder the development of CSCs and initiate deterioration of colonospheres that are very prominent in CSCs [35,36,37]. These discoveries exhibit the utility of CDF as a promising remedial agent for treating several cancers. However, the challenges of poor water solubility of CDF, albeit superior to CMN, has made it difficult for formulating and further for delivering the drug to tumors. In this specific circumstance, we have exploited our findings so far that Bovine Serum Albumin (BSA) could be utilized as a nanoparticles stage for the delivery of CDF [6,38].

### 1.4. Carbonic Anhydrase IX Receptor

The CO_2_ is reversibly converted to bicarbonate by Carbonic Anhydrase (CA), a zinc metalloenzyme thus maintaining the pH of the cells [39]. Among several isotypes of CA, CA IX is one of the highly overexpressed transmembrane proteins in various cancer cells, including TNBC [39,40]. Tumor utilizes CA IX for maintaining acidic pH of the tumor microenvironment to prevent the induction of tumor hypoxia-related cascade reactions and stemness [29,41]. The overexpression in tumor cells compared to healthy tissue where CA IX had limited expression has made it a perfect choice as an important target for tumor treatment [42,43,44]. There are several inhibitors of the CA IX being tested in the clinical trials that show promising results as therapeutic as well as diagnostic agents in solid tumors like the pancreatic cancer and colorectal cancer [45]. These include SLC-0111 (NCT02215850), U-104, G250 (girentuximab) (NCT00087022), Indisulam, DH348, and NIR fluorescent derivative of the acetazolamide. Several of these ligands, such as G250, are at the Phase III clinical trials and have shown good activity in reducing hypoxia induced acidification in the solid tumors and reduce tumor size [46,47,48]. Small molecule conjugates to target CA IX has been on the upsurge in cancer therapy research because of their stability alongside the therapeutic activity [49,50]. Acetazolamide (ATZ) has a high affinity to CA IX [51], and literature has reported that it does not function as a suicidal inhibitor (such as coumarin/thiocoumarin) of CA IX [52,53,54]. It can be further used in conjugation for the diagnosis of various cancers, too [55,56,57].

### 1.5. Hypoxia Targeted Nanoparticles for Better Cell Killing in TNBC

Nanoparticles have been broadly utilized as drug delivery systems for targeted delivery of anticancer drugs in the pharmaceutical industry. They can improve the delivery of hydrophobic drugs, lower their metabolic degradation in the eliminating organs, target their delivery to malignant cancer cells simply by way of modifications to the surface of the delivery systems with the addition of a ligand, and show controlled or extended or sustained delivery of drugs. Polymeric nanoparticles have been broadly utilized as a part of pharmaceuticals as drug delivery systems for targeted delivery of anticancer drugs. They can enhance the delivery of hydrophobic drugs, decrease metabolic degradation of these drugs, target their delivery to malignant cancer cells just by alteration of the surface of the delivery system with the addition of a ligand, and show controlled or extended or sustained delivery of drugs. Besides the capability to improve the solubility of hydrophobic capsules, nanoparticles additionally can target cancer cells with the aid of two distinctive strategies: passive and active targeting. The functions of the passive targeting delivery systems depend on the capacity of nanoparticles to exploit the Enhanced Permeability and Retention (EPR) effect [58,59]. The EPR phenomenon proposes that the multiplication of cancer cells leads to the development of the profoundly disordered blood capillary with leaky tumor vasculature. As a result, the nanoparticles can extravasate and accumulate at the tumor site. Tumors also have poor lymphatic clearance. As a result, the nanoparticles that accumulate in tumor tissues are retained for prolonged periods of time, resulting in continued release of the loaded anticancer drugs. However, the presence of EPR effect is not common to all cancer cells and in some may not be as effective as in the others [60,61]. Furthermore, in those cancers cells that do display the EPR effect, the angiogenesis is not uniform throughout the tumor depth which could cause unequal distribution of the medication. Therefore, the nanoparticles are commonly conjugated with a targeting moiety for promoting cancer cell specific internalization or intracellular organelles that are associated with specific targeting biomarkers in cancer cells. This gives the purpose to develop targeted delivery systems that function via passive and active mechanisms for more selective tumor cell targeting.

**Figure 1:**
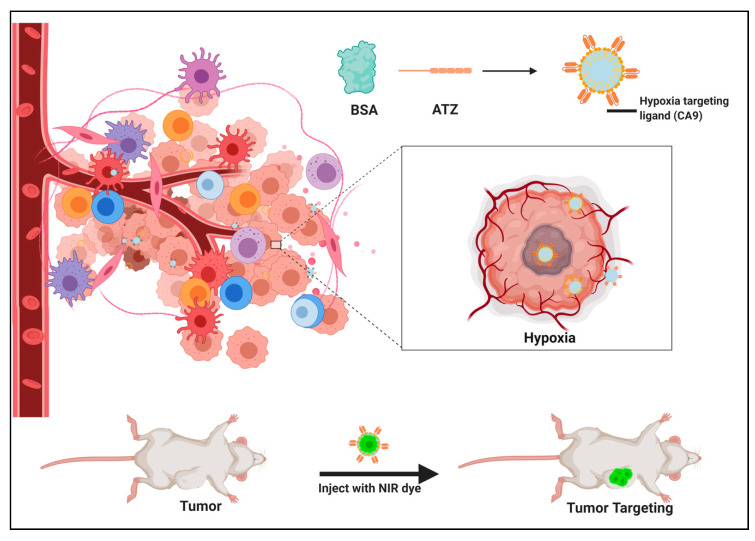
Graphical representation of the research work presented in this paper using the hypoxia targeted formulation comprising of the drug CDF encapsulated in a *click*-conjugated bovine serum albumin and hypoxia targeting ligand, i.e., acetazolamide (BSA-CDF-ATZ) that is used for enhanced cell killing in Triple Negative Breast Cancer (TNBC).

Our study described in this research paper is lined on the concept of the self-assembling BSA nanoparticle encapsulated with CDF and functionalizing the nanocarrier with targeting ligands utilizing strain-promoted copper-free click chemistry. Copper-free click chemistry has several advantages such as (i) environmentally safe, (ii) site specific conjugation, (iii) high yielding and stereospecificity as compared to other chemical conversion (e.g., maleimide-thiol coupling). The click reaction can happen in aqueous conditions, thus, beneficial for nanomedicine and drug delivery purposes. Considering these advantages, we utilize this strategy to conjugate dibenzocyclooctyne (DBCO) functionalized ATZ (ATZ-DBCO) with BSA-N_3_ encapsulated with CDF nanoparticles to target tumor hypoxia. Hypoxia has a role in maintaining the cancer stem cells. The location of the hypoxia is at the core of the tumor, which is relatively unreachable to the treatment modalities and is a major challenge in treating cancers. Thus, our formulation has the potential to establish the proof of concept that the core of the tumor can be targeted as shown in Figure 1, and thus, the efficacy of the therapy can be improved.

## 2. Results

### 2.1. In Silico Screening for the Binding Studies

**Figure 2:**
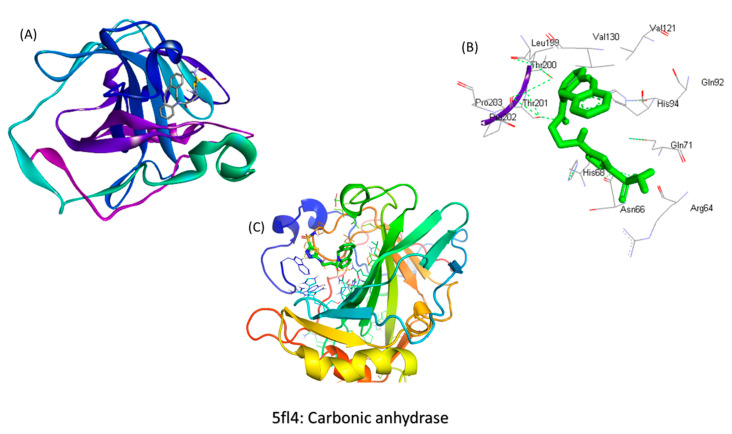
Docking studies for BSA-CDF-ATZ showing the interactions between the ligand ATZ and the macromolecule BSA, and the consequent interaction positions between them. (**A**) indicates the BSA bound to Acetazolamide (ligand); (**B**) indicates the locations where the ligand binds to the CA IX (carbonic anhydrase) receptor; (**C**) indicates the binding of the acetazolamide to the CA IX receptor.

After analyzing the docking interactions (Figure 2A–C), the ligand was found to have the highest binding energy of <−8.4 kcal/mol. It was also found that the ligand was interacting with the CA IX protein at the 1690 position and the amino acids Thr 200, 201 His 68, Asn 66, Gln71, His 71 positions, which also proves our hypothesis that the synthesized ligand was interacting with the Heme group in the hemoglobin and causing its chelation.

### 2.2. Synthesis of the Molecule

**Figure 3:**
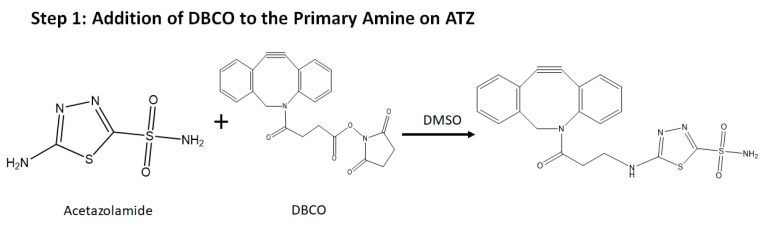

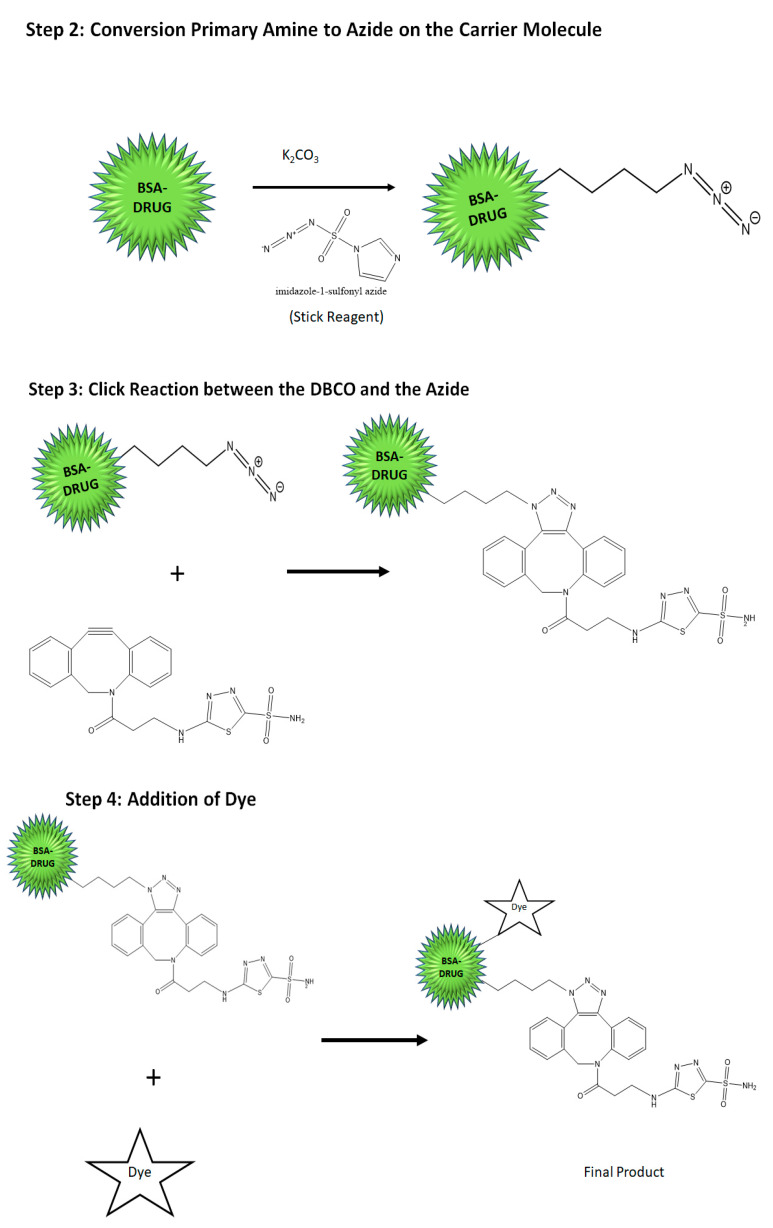
Scheme for the synthesis of the BSA-CDF-ATZ nanoparticle using click chemistry. This scheme illustrates the steps involved in the synthesis of the a theranostic nanoparticle guided by CA IX for cell killing in TNBC.

The synthesis of this nanoparticle follows a procedure of conjugation by click chemistry [62,63,64] (Figure 3). In Step 1, the stoichiometric ratio of the reactants (acetazolamide: DBCO) used are 1:2. In Step 2, the ratio of BSA: Stick’s reagent is 1:5 based on the reasoning that the BSA protein has multiple reacting acidic groups -COOH. In Step 3, the stoichiometric ratio of the DBCO and azide groups are 1:1 because they are complimentary to each other. The product so formed was lyophilized after dialysis to get a powder form and was analyzed by FTIR to confirm the successful progress of the click reaction.

The results of FTIR (Suppl. Figure S1a) demonstrated trademark peaks between 1800–2200 cm^−1^ relating to the ^−^N==N^+^==N^−^ stretch, with a noticeable shift in the peaks confirming the reaction. Peaks were found indicating the presences of -N-H, -C=O, and -C-H bonds at 3300–3500 cm^−1^, 1670–1820 cm^−1^, and 1050–1150 cm^−1^ individually. 1H NMR (Supplementary Figure S1b) showed peaks between 7.2–7.6 ppm that relate to the DBCO’s hydrogens and between 2.2–3.6 ppm demonstrating the azide hydrogen that are separated from the sulfonamides peaks between 7.4–8.0 ppm. Hence, the reaction was reaffirmed.

### 2.3. Characterization

#### 2.3.1. Drug Loading

Drug loading was achieved by the desolvation/coacervation method as described in the supplementary information. The percentage of drug loading was analyzed using HPLC and UV Spectrophotometer at 445 nm for absorbance of CDF and was found to be around 12% w/w for CDF in the nanoparticles. It is our hypothesis that the hydrophobic core comprising of the amino acid groups could be the reason for the high loading of the hydrophobic drug such as CDF.

#### 2.3.2. Particle Size Analysis

**Figure 4:**
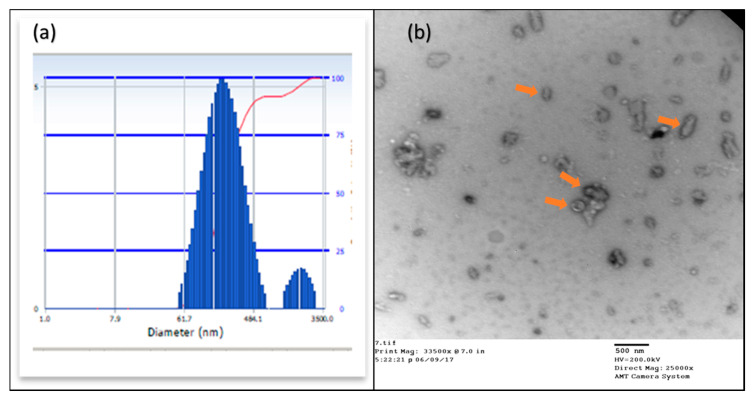
(**A**) DLS of the BSA-CDF-ATZ; (**B**) TEM of BSA-CDF-ATZ. The nanoparticle size from both these analyses was found to be in the desired nanoscale range of below 300 nm for eliciting both active and passive tumor targeting.

The DLS and TEM evaluated the molecule size of the nanoparticles. The results showed that the hydrodynamic particle size (Figure 4A) in DLS was 261.1 nm. The PDI was around 0.135, indicating that by far, a large percentage of the nanoparticles were of the similar nature. Further, TEM was used to understand the structure arrangement and the particle size, which showed particle (Figure 4B) sizes between 18 and 25 nm. This information confirmed that the nanoparticles were within the desired range.

#### 2.3.3. Drug Release Studies

**Figure 5:**
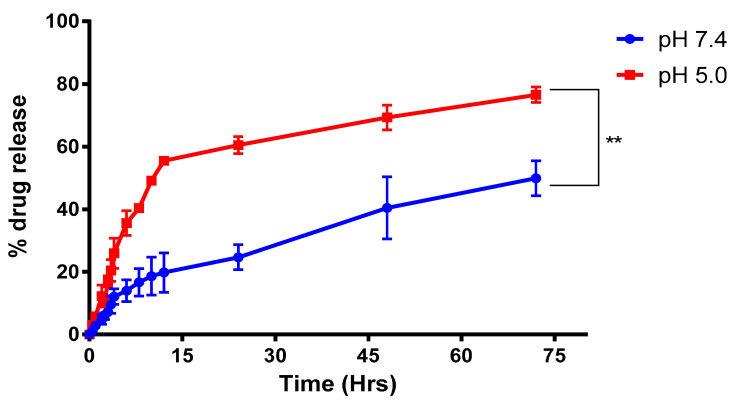
Release pattern observed at pH 7.4 and pH 5.0 for BSA-CDF-ATZ, each representing the normal cell pH and tumor microenvironment, respectively. The higher uptake in the pH 5 confirms better performance and more targetability in the tumor microenvironment in TNBC. Experiments were performed in triplicates; results are shown mean ± SD; **P ≤ 0.01.

The drug release from the NPs was carried out at the physiological pH of 7.4 and pH 5.0, since these relate to that of the blood′s pH and the acidic environment in hypoxic tumor conditions, respectively. The drug release was seen as sustainable over a time of 72 h, which showed around 27.5% at 24 h and 53.8% at 72 h (Figure 5). The mechanism of release is expected to be by a gradual metabolism of the protein carrier BSA. With such trends in releasing the drug from the nanoparticle, the formulation can be classified as a sustained release delivery system.

#### 2.3.4. Shelf-Life Assessments by Stability Studies

**Figure 6:**
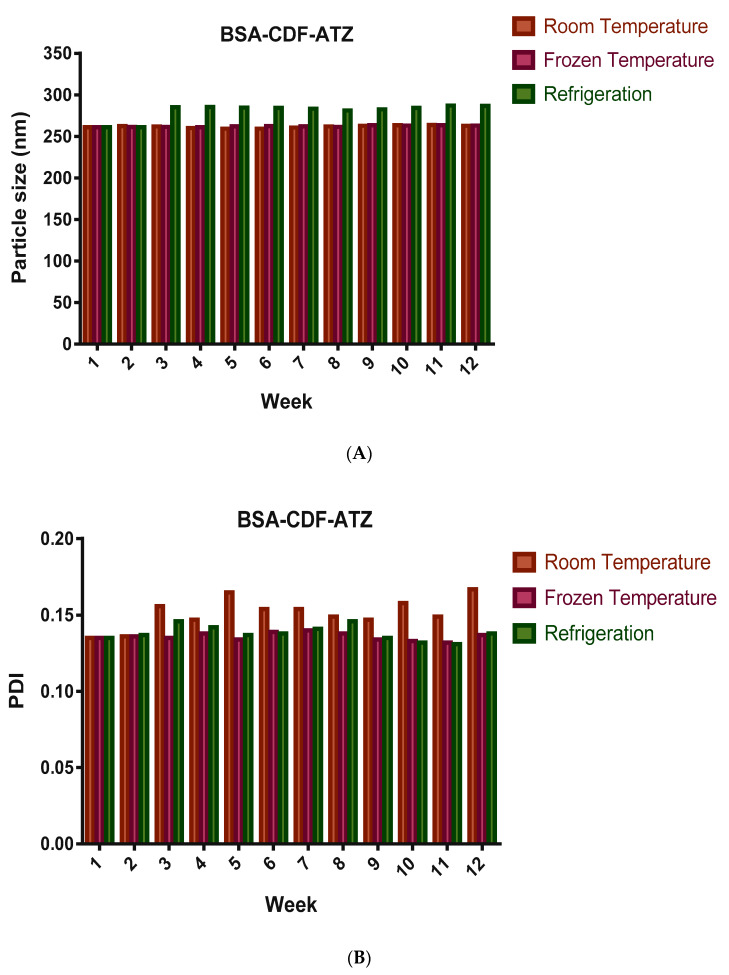

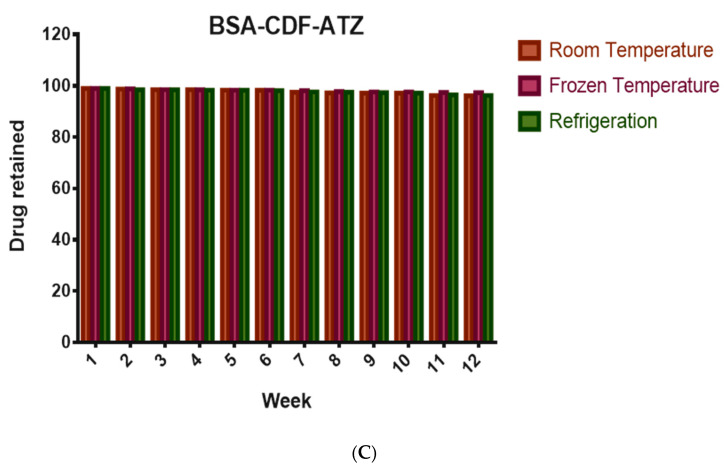
Stability studies after 3 months of storage at different conditions for BSA-CDF-ATZ in terms of (**A**) particle size; (**B**) polydispersity ratio (PDI); (**C**) percentage drug released. There was no significant change in all these characters of the nanoparticles, showing they are stable over a period of time.

These studies are performed to assess the ability of the nanoparticles to resist any leakage of the drug over a long period of time and if there is any agglomeration of the nanoparticles. The stability studies were performed in terms of particle size, PDI, and drug loss over a time of 3 months, and the outcomes are represented in Figure 6A–C. The particle size varied slightly but overall was found to be stable at 262.9 nm at room temperature upon slight shaking. Moreover, it was observed to be steadier in the frozen nanoparticles compared to the other two storage conditions. The PDI was found to be varying too very slightly, and toward the finish of the 3 months was around 0.167. With respect to the drug loss, a 2.8% loss was found following 3 months, when the nanoparticles were stored at room temperature, which was lesser in the frozen conditions at around 1.6%.

#### 2.3.5. In Vitro Cytotoxicity Studies for Understanding the Extent of Cell Killing

The cytotoxic effect of the nanoparticulate formulation was evaluated on two representative cell lines—MDA-MB-231 and MDA-MB-468; each of these cell lines express the CA IX at a different level. It is normal that the MDA-MB-231 presents the receptor to a lesser degree than that of MDA-MB-468 and reacts to a lesser degree to the medicines. Dose response curves were plotted for each of these cell lines for the treatments after analysis using GraphPad Prism 8 as shown in Figure S2a,b. Accordingly, from the cytotoxic studies of the nanoparticle in vitro, an *IC50* at around 31.13 μM of the nanoparticle was calculated for MDA-MB-231 and an *IC50* of 3.78 μM was calculated for the MDA-MB-468 cell line. The outcomes demonstrated a significantly lower effect of the non-targeted and free drug in both the cell lines at every concentration, in comparison to the targeted delivery system.

#### 2.3.6. Comparison of in Vitro Cytotoxicity of the Drug Delivery System in Normoxic and Hypoxic Conditions

**Figure 7:**
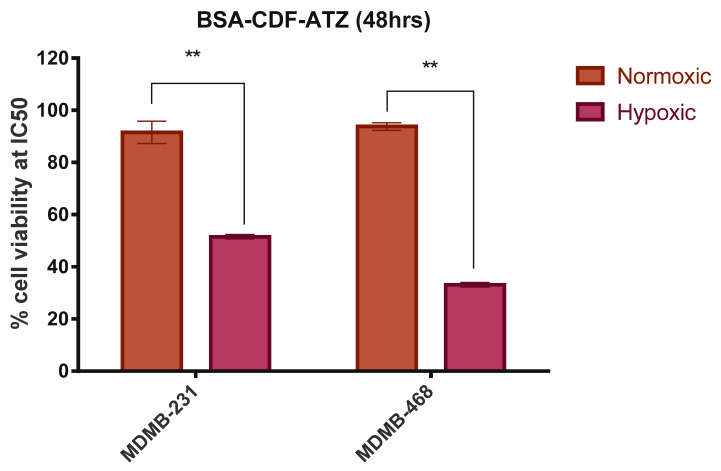
Comparative in vitro cytotoxicity studies for normoxic and hypoxic conditions in MDA-MB-231 and MDA-MB-468 cell lines respectively, for BSA-CDF-ATZ. In both the cell lines, the uptake was higher in the hypoxic condition. Experiments were performed in triplicates; results are shown mean ± SD; **P ≤ 0.01.

The goal of this experiment is to test if the hypoxia-targeted nanoparticle is taken up more and in the hypoxic condition when compared to the normoxic condition. This test is performed by determining their cytotoxicity in either conditions. This test is based on the observation that the CA IX receptor is overexpressed in the hypoxic condition than in the normoxic condition. The utilization of CoCl_2_ actuated the hypoxic condition. The normoxic state was attained when the cells are not treated with CoCl_2_. This was done at the *IC50* value of the drug-loaded NPs in both cell lines (i.e., 31.13 μM in MDA-MB-231 and 3.78 μM MDA-MB-468) and was determined to employ MTT assay. The outcomes of this experiment, as illustrated the Figure 7, show there are more viable cells in the normoxic condition, with the percentage of cells living calculated between 85%–95% and 30%–50% in the hypoxic condition in both cell lines.

#### 2.3.7. Cell Uptake Studies by Fluorescence Spectroscopy

**Figure 8:**
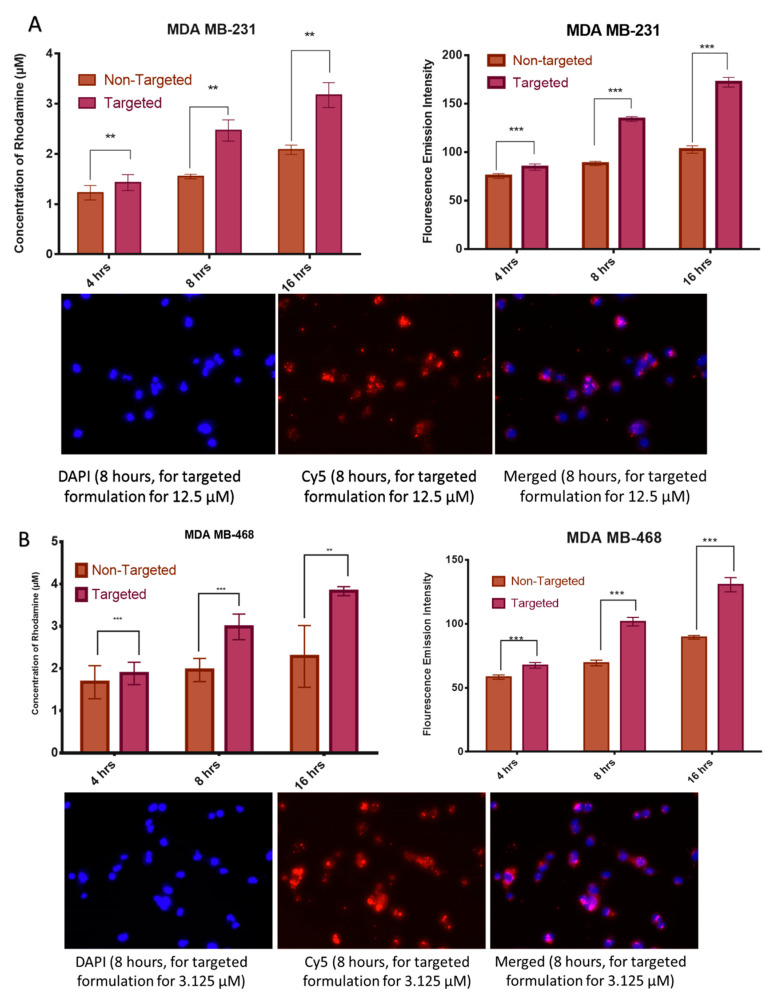
Fluorescence spectroscopic in (**A**) MDA-MB-231 and (**B**) MDA-MB-468 cell lines respectively for BSA-CDF-ATZ. Fluorescence microscopy was done at 8 h for both the cell lines. The uptake of the nanoparticles increased with time. Targeted formulation here indicates BSA-CDF-ATZ and non-targeted formulation indicates BSA-CDF without the hypoxia targeting ligand attached to them. Experiments were performed in triplicates; results are shown mean ± SD; **P ≤ 0.01; ***P ≤ 0.001.

The spectroscopic fluorescence test quantitatively measures the extent of nanoparticles entering into the cells. The nanoparticles conjugated with rhodamine B chemically are used for identification in the spectrometer and the uptake is studied in a time-dependent manner. The higher concentration of rhodamine recognized with time implies a more significant number of NPs encapsulating the drug inside the cells that shows a higher uptake of the formulation in both the cell lines increased. It has also been noticed that the concentration of rhodamine inside the cells increases with time in 4, 8, 16 h (Figure 8A,B). The outcome showed that there is an increase in the rhodamine intensity with time. Similar trends were observed in the fluorescence microscopy.

#### 2.3.8. Extent of Apoptosis Studied by Flow Cytometry

**Figure 9:**
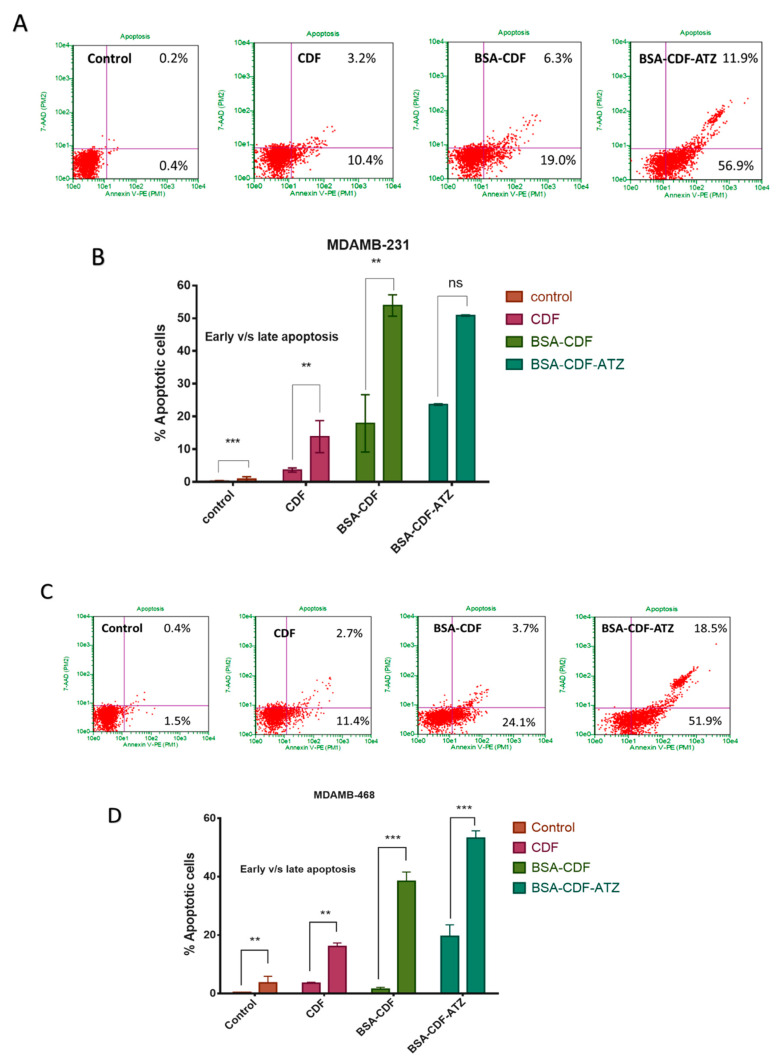
Apoptosis assay studies and their graphical interpretations in (**A**,**B**) MDA-MB-231; (**C**,**D**) MDA-MB-468 for BSA-CDF-ATZ. The targeted nanoparticles have illustrated a better cell killing in the tumor cells in both cell lines. Experiments were performed in triplicates; results are shown mean ± SD; **P ≤ 0.01; ***P ≤ 0.001.

We hypothesize that the treatment with the targeted nanoformulations induces apoptosis in both the TNBC cell lines. This hypothesis is tested using flow cytometry with Annexin V/7-AAD dual staining. The level of Annexin V^+^/7-AAD^+^ (R3), Annexin V^−^/7-AAD^+^ (R4), Annexin V^−^/7-AAD^−^ (R5), and Annexin V^+^/7-AAD^−^ (R6) were calculated as representative of the percentage of live cells, early apoptotic, late apoptotic and necrotic cells, respectively. In the MDA-MB-231 cell line, the apoptosis was observed to be 56.9% (early apoptosis) and 11.9% (late apoptosis), respectively, when treated with targeted formulation treated cells and 19.0% and 6.3% respectively with non-targeted formulation (Figure 9A). Similarly, in the MDA-MB-468 cell line, the targeted formulation showed 51.9% and 18.5% of early and late apoptosis, respectively; the non-targeted formulation showed 24.1% and 3.7% (Figure 9C). These outcomes present the superior apoptosis-inducing capacity of the targeted formulation when compared to non-targeted formulation. The graphical representation of these results has been demonstrated in Figure 9B,D.

#### 2.3.9. In Vivo TNBC PDX Tumor Targeting Efficacy in Mice

**Figure 10:**
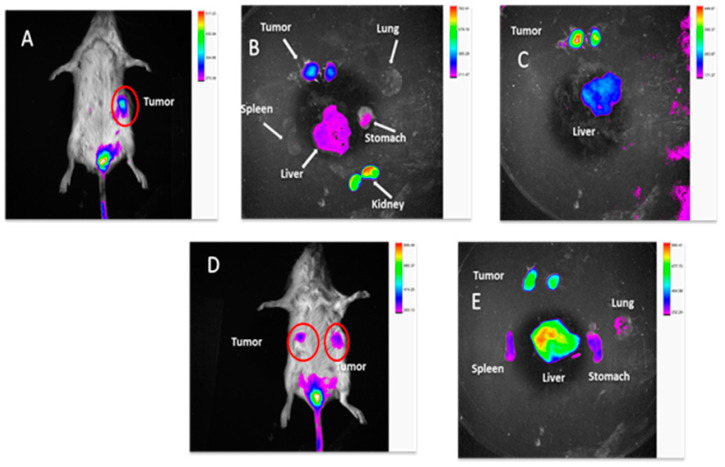
The tumor hypoxia specific imaging of BSA-CDF-ATZ-S0456 in TNBC patient derived tumor xenograft (PDX) Nod/SCID mice. (**A**) The extent of tumor targeting via hypoxia in mice; (**B**) The bio-distribution of the targeted formulation in the organs; (**C**) Comparative bio-distribution of the targeted formulation in liver and tumor; (**D**,**E**) the extent of tumor targeting via hypoxia for the non-targeting drug delivery system BSA-CDF in mice and the bio-distribution respectively.

Near-infrared (NIR) imaging has the advantages of cost-effectiveness and less toxicity, and hence, can be described as an advanced diagnostic tool for tumor detection. TNBC demands early detection because it is asymptomatic with a high rate of metastasis to assist with either pharmacological or surgical treatments. We hypothesize that our nanoparticle can be a valuable tool to achieve this goal. To prove the in vivo targeting efficacy of BSA-CDF-ATZ nanoparticles in treatment of TNBC, we conjugated the S0456 near infrared dye that has clinical relevance and structural advantages for chemical conjugation (BSA-CDF-ATZ-S0456). It can be seen from the results that the targeted formulation is localized in the tumor that is overexpressing CA IX after 24 h (Figure 10A–C) compared to the non-targeted formulation (Figure 10D,E) post single i.v. injection. Major organs/tissues were separated for ex vivo analysis of the efficacy of the nanoparticles to understand the bio-distribution at 24 h post-injection for both the targeted and non-targeted formulations. An interesting observation from this study is that there is very little uptake of the formulation seen in the liver and spleen, resolving the major clinical problem of nanoparticles that extensively accumulated in liver. Furthermore, some uptake of NIR dye in kidneys indicated the kidney-mediated excretion. These results indicate that the CA IX receptor targeting of BSA-CDF-ATZ nanoparticles can selectively accumulate in tumor with reduced liver and spleen uptake.

#### 2.3.10. Statistical Analysis of the Results of Cytotoxicity Studies

Statistical analysis using Student’s t-test was performed to calculate the significance of the difference of treatment between the two cell lines, hence, comparing the efficiency of the formulation in both the cell lines. The *p*-value was found to be 0.00274, which proves that there is a significant difference in the response between the two cell lines to the formulation. Furthermore, both the cell lines showed a significant difference between the efficiency of the targeted and free drug, with *p*-values of 0.003046 in the MDA-MB-468 cell line and 0.003862 in MDA-MB-231 cell line. In the cell uptake studies performed on this formulation, the *p*-value of <0.05 indicated a significant difference in the extent of uptake of the non-targeted and targeted formulations. A *p*-value >0.05 for the parameters of the stability studies shows no significance in the difference emphasizing the stability of the formulation.

## 3. Discussion

The essential goal of the targeted delivery system is to improve a nanodelivery framework to address the difficulties experienced in the delivery of drugs to particular targets identified for a disease condition [65,66,67]. It is an additional advantage to design it using a reagent free click chemistry process because of the apparent ease of applicability of the technique. Recently, click chemical reactions have proven to be very promising for developing drug delivery systems. This unique reaction technique depends on the strain promoted functional groups, such as DBCO and Azide. The increasing use of this reaction technique has demonstrated the benefits for the pharmaceutical sciences field, drug delivery, and biomaterials synthesis [68]. Click reactions can be of several types that include (i) cycloaddition of unsaturated species like 1,3-dipolar cycloaddition, (ii) cycloaddition of unsaturated species like [4+2]-cycloaddition (Diels–Alder), nucleophilic substitution/ring-opening reactions, non-aldol carbonyl reactions [69]. The advantages of click chemistry have been gaining more importance in the discovery, delivery, and bioconjugation of the drugs [70]. Copper-free click reaction is a simple, rapid, and highly selective, water-compatible, and bio-orthogonal chemical approach that involves ligation of two moieties through strain-promoted 1, 3 dipolar azide-alkyne cycloadditions [71,72]. Copper-free click reactions are preferred to the copper-catalyzed click reactions since copper-free reactions do not involve the oxidative damage and toxicity that could be caused to the cells and tissues due to residual copper catalysts remaining in the purified product [73]. The driving force for the copper-free click approach is the strain of the alkyne encountering ring, as in the dibenzyl cyclooctyne (DBCO) ring instead of the copper catalyst in the copper-catalyzed reaction [72]. The copper-free click reaction reported by Bertozzi and co-workers is the biocompatible and bio-orthogonal that are observed in living systems albeit toxicity or interference with native biological molecules or processes [71,74]. Here, we utilized a copper-free click reaction that provides several advantages, such as (i) feasibility of conjugating the CA IX targeting ligand, ATZ, (ii) no need to perform any further purification, (iii) preserving nanoparticle natures. Thus, the safety of the nanoformulation system was maintained using the copper-free click reaction technique.

To achieve this goal, the first step for us was to determine if the ligand Acetazolamide was able to bind to the CA IX receptor. The in silico studies have given a great insight about the binding energy of the ligand to the receptor and the specific positions of binding. We confirmed that the interaction between the ligand and the receptor is present and the strength of this bond is very strong. Once the efficiency of the ligand had been established thus, it was confirmed that Acetazolamide could be used for targeting CA IX that is overexpressed in hypoxia. The next step was to synthesize the targeted formulation using click chemistry. The physicochemical characterization of BSA-CDF-ATZ was performed employing TEM, DLS that showed nanoparticulate nature of the particles, and narrow polydispersity index that demonstrated the homogenous particle distribution. These signature characteristics and having high-affinity CA IX targeting ligand of BSA-CDF-ATZ promotes the infiltration of the nanoparticles into the tumor through two mechanisms of EPR effect and active targeting, respectively [3,4]. This targeted uptake of the nanoparticles into the tumor in conjunction with the results of TEM and DLS can explain the cytotoxic effect eventually. The BSA-CDF-ATZ nanoformulations maintained sustained drug release properties and maintained a low polydispersity index over a tested period of 3 months. BSA-CDF-ATZ was found to be robust with minimal drug loss for a period of stability studies. This means that the long period of drug availability is achieved through the formulation. Further, the stability of the NPs was also tested by storing the NPs at freezing conditions temperatures without any significant drug loss.

MDA-MB-231 is pre-epithelial to mesenchymal breast cancer cell with high metastatic and invasive property, and MDA-MB-468 is basal breast cancer cell [75]. The significantly higher cytotoxicity of BSA-CDF-ATZ, when compared to BSA-CDF or CDF in both the cell lines used for this work, suggest the broad application of CA IX targeting BSA nanoparticles in various TNBC tumor types [76]. Tumor hypoxia is prevalent at low pH condition that ultimately assists the proliferation of tumors, with higher expression of CA IX, and increases the stemness of cancer [77]. Thus, BSA-CDF-ATZ based NPs are being taken up more efficiently in the TNBC cell line [78,79]. The cell viability difference under normoxic and hypoxic conditions under CA IX-targeted formulation treatment strengthen the hypothesis that the nanoparticles are accumulating by a specific CA IX receptor-mediated uptake mechanism [80,81,82]. Furthermore, the high fluorescence uptake of Rhodamine-labelled BSA-CDF-ATZ compared to non-targeted and free Rhodamine is a strong indication of CA IX receptor-mediated uptake in the cells.

The data suggested higher cytotoxicity of BSA-CDF-ATZ in hypoxia conditions compared to normal oxygen condition, and more prominent uptake of Rhodamine labeled BSA-CDF-ATZ demonstrates that CA IX is an essential marker of TNBC that can further be exploited for the development of targeted therapy of TNBC [11,66,68,83,84]. In addition, to illustrate the mechanism of cell killing, we performed an Annexin-V/7-AAD dual stained-based apoptosis assay of the TNBC cells. The increment of both early and late apoptotic cell population in BSA-CDF-ATZ treated cells as compared to other treatments strengthened the hypothesis of CA IX directed programmed cell death that is linked to the pathway of apoptosis.

The in vitro data have been positively corroborated by the in vivo tumor imaging of the CA IX-formulation in the PDX-breast cancer model. The objective of this research was to determine the targetability of the formulation in the human tumor mimetic TNBC model. The results were promising in showing that the BSA-CDF-ATZ formulation is successful in selectively targeting cancer with a minimum uptake by non-target tissues and also that the formulation has limited uptake in the liver and spleen. The possibility of drug toxicity due to accumulation in these organs can be evaluated in a separate set of toxicological studies. These observations ascertain that the formulation is promising in delivering cytotoxic payload for effective antitumor therapy study and tumor immune modulation. It has been already established that the advancement of optical image-guided surgery has increased the importance of fluorescent NIR dyes used to differentiate malignant from healthy tissues. In clinical trials, there is a critical advancement in the usage of targeted NIR imaging agents that bind specifically to the EGFRs for head and neck tumor (NCT01987375), folate receptor for ovarian (NCT02317705), and PSMA for prostate cancer (NCT02048150) respectively. There are suggestions of reports in the literature on the availability of targeted NIR dye in the clinical trial. Thus, our tumor selective approach of using BSA-CDF-ATZ-S0456 nanoparticles for imaging purposes creates an opportunity for the development of NIR-imaging agents that are very selective for image-guided intra-operative TNBC surgery. In this manner, the present study sets up the dual methodology of NIR-imaging in preclinical tumor xenograft models specific to TNBC to assess the cytotoxic impact of CA IX receptor-targeted CDF-loaded nanoparticles. This research study establishes a proof-of-concept for the developed nanoparticulate delivery system as an effective strategy for the delivery of drugs/dyes via the CA IX biomarker [85,86,87,88,89].

## 4. Materials and Methods

The details for the materials used for this research work are provided in the Supplementary Information file. The methods are detailed here:

### 4.1. In Silico Screening for the Binding Studies

• Retrieval of atomic structure and analog preparation:

All the software used for the analysis are freely available for academic use. The PDB (www.rcsb.org) [56] is a worldwide repository for processing and distribution of 3D biological macromolecular structure data. The protein structure of Carbonic anhydrase IX (CA IX) (PDB ID 5fl4) was downloaded from PDB.

• Drug targets:

The structure of ligand was prepared in ChemDraw 12 software and modified to PDB file format in DSV which was further used for docking studies.

• Molecular docking (MD):

In silico, MD approaches have broadly been used to know the binding mode of ligand to protein and its application for drug discovery. First, find the CA IX protein binding site identified by Q-SiteFinder. The energy was minimized for ligand and was docked into the cavity site of the protein, using Autodock (version 4.2), which is a set of automated tools. The present study was confined to MD and was performed to calculate the extent of drug-receptor binding energy [57,58].

• Making of the files for the docking studies:

The PDB files were obtained from the Protein data bank (rcsb.org) and modified for the docking study. Using the GUI of ADT, files were prepared. For the AutoDock process, the files are needed in a specific pdbqt format, which is a modified protein data bank format having atomic charges, atom type definitions, and for ligands, all the topological information. These file preparations are carried out by the plugin using scripts from the AutoDock Tools package.

• The Docking Parameters: 

The auto-grid box of dimensions 48 × 34 × 40 Å with the XYZ directions and with a grid point spacing of 1Å was established by using the Auto-Grid module to predict the bound conformation based on the empirical force filed Lamarckian Genetic Algorithm (LGA) runs performed with both molecules and gives free binding energy. Docked ligand binding site analysis performed using PyMOL and DSV. Finally, Ligplot software was employed to analyze the 2D structure of the final docked structure.

### 4.2. The Synthesis of the Hypoxia Targeting System

The strategy of synthesis of the targeted delivery system designed to bind to the CA IX overexpressed in hypoxic condition in the tumor in this study is a basic four-stage click chemistry reaction. As specified before, click chemistry is a straightforward and fast procedure of coupling two molecules having favorable functional groups that simply click with each other with the simplest reaction conditions. Specifically, the copper-free cyclic Alkyne-Azide click reaction was applied for this research work. This convenient reaction procedure and has been used to create of a library of ligands and carrier moieties with favorable functional groups that can react in click reactions and hence can be made based on the requirement of the disease and the patient in an instant. The chemical scheme of this reaction procedure is shown in Figure 1 and is described briefly below (the detailed procedure is given in the Supplementary Information file):

(a) Synthesis of the targeting ligand: Acetazolamide is the ligand bound on the BSA particle. The amide group of the acetazolamide is first activated to a primary amine group. This activated amine is conjugated to DBCO that has the functional group for click chemistry.
(b) Preparation of the nanocarrier: Nanocarrier is prepared by the converting the amine group of the amino acids present on the BSA protein to azide group utilizing the Stick Reagent (imidazole-1-sulfonyl azide) and K2CO3 overnight.
(c) Drug loading into the carrier molecule containing the azide group: The drug is loaded by desolvation technique utilizing methods that are previously described in the literature [31,32,33,34].
(d) Conjugation of the ligand to the carrier molecule: In this step, the conjugation of the alkyne group on DBCO and the azide group on the carrier molecule with each other in a click reaction is performed at a pH of 8 and room temperature for 4–6 h. The final product is water-soluble. The product can likewise be conjugated further with an NIR dye by the click reaction or simple conjugation under stirring to result in a theranostic product.

### 4.3. Characterization

#### 4.3.1. Drug Loading

The method used to load the drug into the nanoparticle is described in Section 4.1. and in detail in the Supplementary Information file. The quantitative measurement of drug loading was done using HPLC and UV Spectrophotometer, and the quantity of drug encapsulated was calculated from the standard graph developed from both the HPLC and UV Spectrophotometer. Detailed information is provided in the Supplementary Information file.

#### 4.3.2. Particle Size Analysis

The nanoparticles were additionally assessed in terms of particle size utilizing a Beckman Coulter Delsa Nano-C DLS Particle analyzer (Beckman Coulter, Inc., Fullerton, CA, USA) that included a 658 nm He-Ne laser as described before by our lab. The detailed information is given in the SI file.

#### 4.3.3. Drug Release Studies

Analysis of the amount of drug released from the nanoparticle was done with the final product prepared as described in the Section 4.1. This is usually done in normal physiological conditions and also in the simulated tumor microenvironment in terms of pH. The detailed methodology is provided in the Supplementary Information file.

#### 4.3.4. Shelf-life Assessments by Stability Studies

The stability of the formulation over the shelf life period was evaluated in terms of particle size, PDI, and drug loss at three distinct conditions, i.e., at room temperature (25 °C), refrigeration (4 °C), and freezing conditions (−20 °C) for 3 months [12,36]. The samples were extracted each week and investigated for the said parameters.

#### 4.3.5. Cell Culture

MDA-MB-231 and MDA-MB-468, both of which correspond to Triple-Negative Breast Cancer (TNBC), were the used as the target cell lines to test the final formulation [54,55] because of their expression level of the hypoxia marker (CA IX) receptor on the surface of the cell lines has been previously determined in the literature. Further details are provided in the Supplementary Information file.

#### 4.3.6. In Vitro Cytotoxicity Studies for Understanding the Extent of Cell Killing

The in vitro cytotoxicity of the formulation was calculated upon induction of hypoxia utilizing CoCl_2_. This treatment results in the high expression of the CA IX receptor on the surface of both the cell lines. Detailed information is provided in the Supplementary Information file.

#### 4.3.7. Comparative in Vitro Cytotoxicity Studies for Normoxic and Hypoxic Conditions

Tumorous cells demonstrate a distinction regarding the presence of hypoxia and the consequent acidity around them. Though the tumor cells in normoxia express the CA IX receptor to a small extent, it is not to the extent found in the tumorous cells in hypoxia. Hence, this study was performed to comprehend the distinction in the uptake of the targeted formulation in the normoxic conditions and in the hypoxic conditions in both the cell lines. Detailed information is given in the Supplementary Information file.

#### 4.3.8. Cell Uptake Studies by Fluorescence Spectroscopy

The uptake studies were carried out employing fluorescence spectroscopy as well as fluorescence microscopy. The detailed methodology for the uptake studies is provided in the Supplementary Information file.

#### 4.3.9. Apoptosis Assay by Flow Cytometry

MDA-MB-231 and MDA-MB-468 cell lines were subjected to apoptosis assay separately as per the literature. The detailed methodology for this test is provided in the Supplementary Information file.

#### 4.3.10. In Vivo TNBC PDX Tumor Targeting Efficacy in Mice

Female tumor-bearing Nod/SCID mice (6–8 weeks old) were purchased from Jackson Laboratories and were housed in a sterile environment on a standard 12 h light-dark cycle and maintained on normal rodent diet and water. All animal procedures were approved by the Wayne State Animal Care and IACUC committee following the National Institutes of Health guidelines. Further information is provided in the Supplementary Information file.

#### 4.3.11. Statistical Analysis of the Results of Cytotoxicity Studies

To understand the significance of the difference between the response to the formulation between the two cells and also the importance of the difference between the responses to the free drug and the formulation in each cell line, statistical analysis using the t-test was done to calculate the *p*-value.

## 5. Conclusions

Hypoxia is a condition that is pervasively present practically in all tumors. In any case, the quickly developing malignant cells produce an absence of oxygen, bringing down the pH which will bring about the overexpression of the Carbonic anhydrase IX receptor. CA IX is a very extensively discussed biomarker in light of hypoxia. The expression of CA IX in numerous tumors shows its pertinence as a suitable marker of hypoxia in many tumors. Additionally, its appearance is firmly identified with the projection of the clinical outcomes in various tumors. This research emphasizes this idea, which can be accomplished employing ligands like Acetazolamide and a biologically safe transporter-like BSA that are readily available for clinical use and henceforth do not elicit adverse immune responses. The results of this study show that the tumors are explicitly taking up the nanoparticles, leading to much less viable malignant cells and showed a high level of apoptosis in them. The outcomes further demonstrated that the ATZ based framework is very effective to be utilized for drug delivery. The delivery of the drug through the developed NPs showed a very high specificity towards the cells. The conjugation of specific dye further makes it a theranostic framework. With the supporting data, we can further use CA IX targeted NPs for in vivo therapeutic purposes as well.

## Supplementary Materials

The following are available online, Figure S1a: FTIR for BSA-CDF-ATZ; Figure S1b: NMR for BSA-CDF-ATZ; Figure S2: In vitro cytotoxicity studies in (a) MDA-MB-231 and (b) MDA-MB-468 cell lines; S1: Materials; S2: Methods in detail.
