# Supplementary material for: Carbonic Anhydrase-IX Guided Albumin Nanoparticles for Hypoxia-mediated Triple-Negative Breast Cancer Cell Killing and Imaging of Patient-derived Tumor
Source: Molecules. 2020 May 19;25(10):2362. doi: 10.3390/molecules25102362 (PMC7287925; doi:10.3390/molecules25102362)
Supplement: Supplementary file 1 [file molecules-25-02362-s001.pdf]

Carbonic Anhydrase-IX Guided Albumin Nanoparticle for Hypoxia Mediated Cell Killing and Imaging of Patient Derived Triple Negative Breast Cancer

Katyayani Tatiparti1, Mohd Ahmar Rauf1, Samaresh Sau1, Arun K. Iyer1, 2,*

Supplementary Information

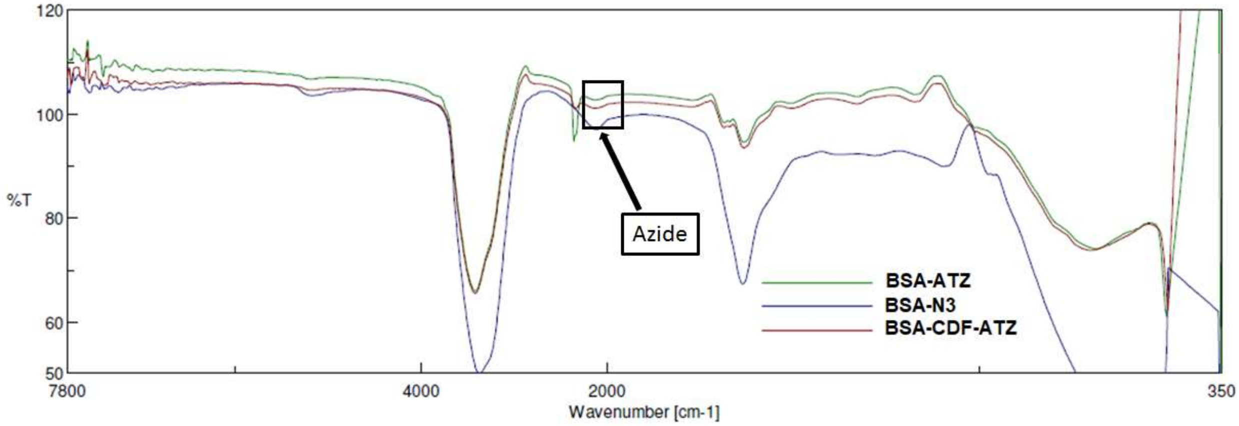

Fig. S1a: FTIR for the hypoxia targeting drug delivery system BSA-CDF-ATZ

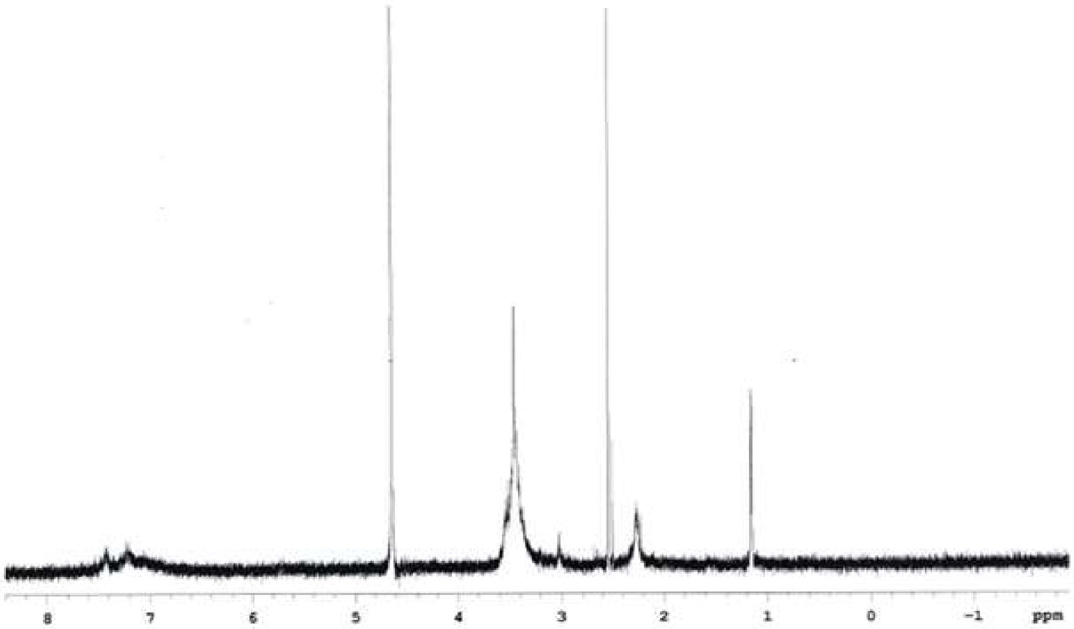

Fig. S1b: NMR spectrum for the hypoxia targeting drug delivery system BSA-CDF-ATZ

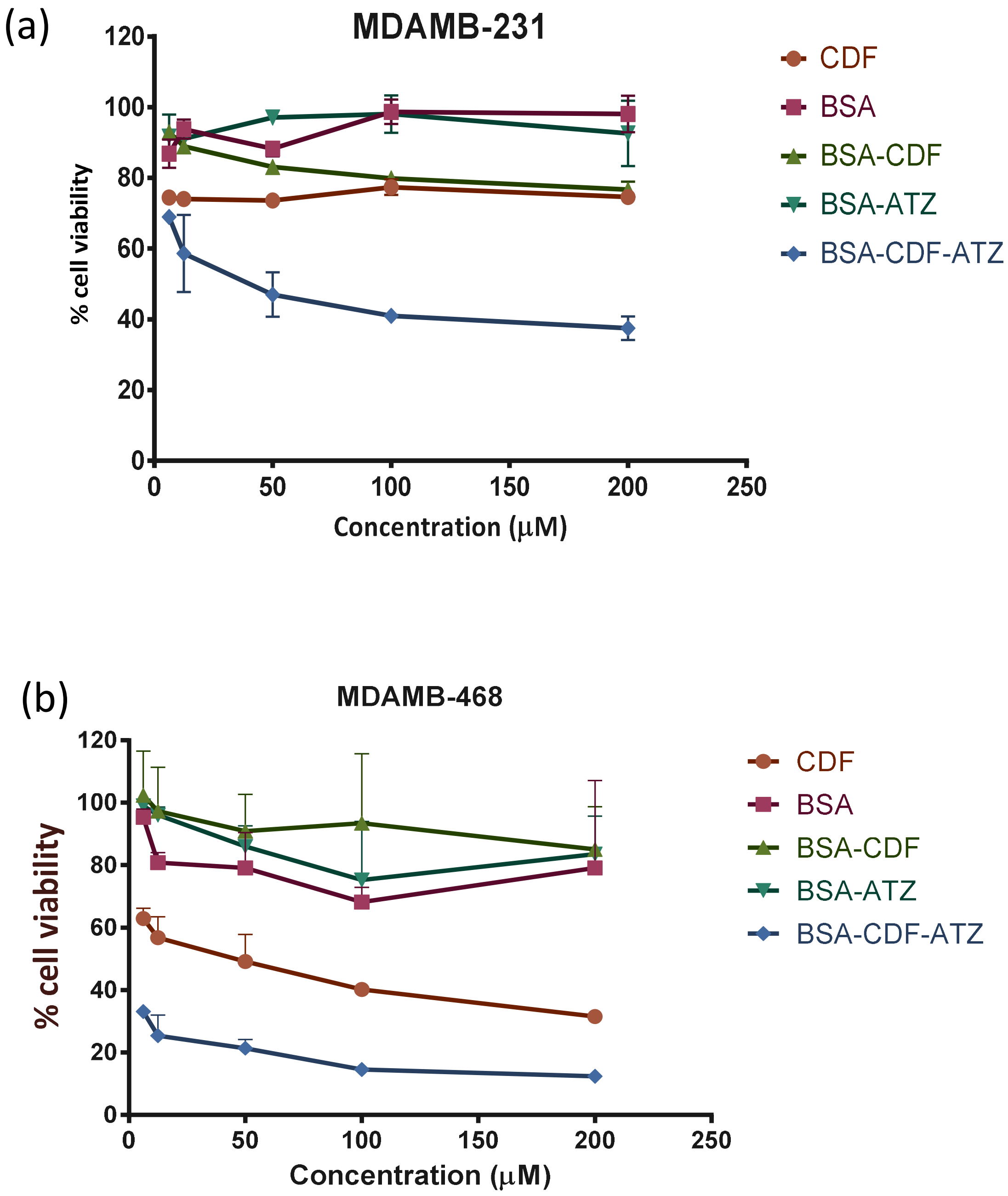

Fig. S2: ***In-vitro*** **cytotoxicity studies in (a) MDA-MB-231 and (b) MDA-MB-468 cell lines respectively for the tested compounds**

1. Materials

Protease-free bovine serum albumin (BSA) was procured from LEE BiosolutionsTM. Paclitaxel (PTX) was purchased commercially from LC Laboratories. DBCO was procured from Click Chemistry Tools Bioconjugate Technology Company (Scottsdale, AZ, USA). Stick's reagent, i.e., imidazole-1-sulfonyl azide hydrochloride reagent, was synthesized according to the previously reported method [29] was prepared in the laboratory. Potassium carbonate was purchased from Fisher Science Education (Nazareth, PA, USA). Dimethyl sulfoxide (DMSO) 99.7% was purchased from Acros Organics. Glutaraldehyde solution (50% in water), EDC, and NaCl (99.0% pure) were purchased from Sigma-AldrichTM. Annexin V reagent kit was purchased from EMD Millipore Corporation. DMEM, life cell fluorescence imaging medium, Phosphate-buffered saline (PBS), fetal bovine serum (FBS), penicillin-streptomycin (Pen-Strep), and Trypsin-EDTA (0.25%) with phenol red solution were from Gibco, Thermo Fisher Scientific (Waltham, MA, USA). MDA-MB-231 and MDA-MB-468 cell lines were obtained from collaborators. All solutions were prepared using pure deionized water.

2. Methods

3.1. Synthesis and Characterization of the Hypoxia Targeting Drug Delivery System

The synthesis of the drug delivery system developed in this study that targets hypoxia in the tumor cells follows a simple four step reaction using click chemistry. As cited before, click chemistry is a quick technique of conjugating molecules which have complimentary functional groups that just 'click' with each other in minimal conditions of the reaction. The precise form of click reaction used in this study is the copper free cyclic Alkyne-Azide click reaction. The ease of this reaction technique allows the designing of a library of ligands and carrier molecules that have those complimentary molecules that can take part in click reaction that may be made on a need-basis. The scheme of this reaction is provided in the Figure 1. The outline of this procedure is as follows:

Preparation of the targeting ligand: Step one of the procedure is to design the ligand such that it can be added onto the BSA molecule. The reactant molecule is the Acetazolamide. The amide group of the acetazolamide is first transformed to primary amine by using acid hydrolysis utilizing 1M HCl. The primary amine group hence activated is then used to be connected to one of the complimentary molecule, DBCO with a $-C\equiv C-$ group that similarly participates within the click reaction. The acetazolamide with the primary amine group and the DBCO are dissolved in DMSO and then allowed to react for overnight at room temperature whilst stirring constantly. The product is then dialyzed in a 12 kD dialysis bag (Spectrapor, Spectrum Research Facilities, SD) for 4–8 h and kept readily equipped for further conjugation.

Preparation of the carrier molecule: Preparation of the carrier molecule is the subsequent step. This includes conversion of the amine group of the amino acids comprised on the BSA protein to azide group. This involves two steps. First, the amine groups are transformed to azide groups, $^{-}N=N^{+}=N^{-}$, with the usage of the Stick Reagent (imidazole-1-sulfonyl azide) in the presence of K_2CO_3 overnight at room temperature and non-stop stirring. The addition of these two components of the reaction is carried out on ice because they are exceptionally exothermic reactions. After the conversion, the azide product is dialyzed in a 12 kD dialysis bag (Spectrapor, Spectrum Research Facilities, SD) for 4–8 h and the drug is loaded onto it.

Drug loading on the carrier molecule comprising the azide group: The drug loading is completed by using desolvation technique that is derived from the coacervation procedure using formerly described technique within the literature [30–33]. Paclitaxel is a hydrophobic drug which is initially dissolved in ethanol. BSA was in the beginning completely dissolved in phosphate buffer made in deionized water (50 mg/mL) and positioned on a stirrer at a velocity of around 600 rpm. The pH of the buffer is maintained at 8. The ethanolic solution of the PTX is then introduced into the BSA solution specifically at rate of 1 mL/min. The pH is once more maintained at 8 with the usage of the same buffer. After half an hour of stirring, 8% glutaraldehyde is introduced into the reaction mixture to promote crosslinking within the BSA for encapsulating the drug successfully. The reaction is continued overnight whilst stirring at room temperature. The product is then dialyzed in a 12 kD dialysis bag (Spectrapor, Spectrum Research Facilities, SD) for 4–8 h. This drug loaded carrier molecule with the azide group contributes the other complimentary molecule of the click reaction.

Conjugation of the ligand and the carrier molecule: This is the very last step of the procedure of synthesis of the hypoxia targeting drug delivery system. It entails the conjugation of the alkyne group on DBCO and the azide group on the carrier molecule that click conjugate with each other. This is the simplest step of the method where in the ligand containing the DBCO and the carrier molecule with the azide are combined together in a reaction at pH condition of 8 and at room temperature whilst continuously stirring at 600 rpm. The reaction is performed for 4–6 h. The final product is dialyzed in a 12 kD dialysis bag (Spectrapor, Spectrum Research Facilities, SD) for 2 h and lyophilized. The final product is water soluble. The product also can be attached further with an NIR dye by using the same click reaction to provide a theranostic product that is further taken up for characterization studies using FTIR and NMR studies to affirm the reaction.

3.2. Drug Loading

The process of drug loading was achieved at room temperature as defined earlier within the synthesis Section 3.1.1. The calculation of percentage of drug loaded was accomplished in HPLC and UV Spectrophotometer and the amount of drug encapsulated was determined using the standard graph previously developed by the techniques laid out in literature for PTX in both the HPLC and UV Spectrophotometer. A series of dilutions had been made for the pure PTX and the absorbance for each was taken at 227 nm. A standard graph was plotted according to the readings. Later, a certain amount of the product was taken and examined for absorbance. The quantity of drug encapsulated from the line equation obtained from the standard graph.

3.3. Particle Size Analysis

The nanoparticles were taken for particle size studies using a Beckman Coulter Delsa Nano-C DLS Particle analyzer (Beckman Coulter, Inc., Fullerton, CA) that comprised of a 658 nm He–Ne laser as mentioned earlier by our lab. Tests had been additionally examined with the aid of JEOL Transmission Electron Magnifying tool outfitted with LaB6 filament gun (JEM 2010, Tokyo, Japan) at an accelerating voltage of 200 kV for studying the particle morphology [1–3]. The nanoparticles have been additionally depicted for surface morphology by means of transmission electron microscopy (TEM). tests were set up as described in the previous literature. A determined amount of every sample (4 µl) was applied to a Formvar-coated, carbon stabilized copper matrix (400 work). The copper matrix was air-dried, stained negatively with 5% aqueous uranyl acetate, and was left to dry.

3.4. Drug Release Studies

Drug release studies had been accomplished with the drug delivery system to assess the extent to which the drug has been released out from the system. Preferably, this is executed within the physiological conditions as well as the acidic condition found in the tumor microenvironment. For this research, the studies were executed at pH 7 and pH 5. The evaluation was performed at room temperature. A set quantity of the formulation was placed in a dialysis bag in the sink condition and was continuously stirred. Specific volumes of samples had been extracted at 2, 4, 8, 10, 12, 24, 48, and 72 h from the dialysis bag. The nanoparticles were subjected to high shear in a sonicator for about 15–30 min to disrupt the nanoparticle formation so that the whole drug is released out after which the mixture was centrifuged at high speed of 15,000 rpm for 15 min. This formed a pellet of the disrupted BSA (being a heavy molecule in comparison to the drug) at the bottom and the supernatant obviously contained the drug. The supernatant was extracted, diluted and then analyzed for drug content in UV and HPLC (Waters Corp, Milford, MA) [1,4,5]. The amount of drug was calculated from the line equation acquired from the standard graph which was then subtracted from the amount within the sample first taken to get the quantity of drug released.

3.5. Stability Studies

The stability studies were performed to check for the stability of the formulation over a period of time in terms of particle size, PDI, and drug loss. The formulation prepared was stored at three different conditions of temperatures, i.e., at room temperature (25 °C), under refrigeration (4 °C), and under frozen conditions (−20 °C) for 3 months [6,7]. In the frozen condition, each time the same formulation was used each time so that it could be tested under freeze-thawing condition too. The samples were retrieved every week with, and analyzed for the particle size, PDI in DLS, and drug content in HPLC and UV.

3.6. Cell Culture

The cell lines chosen for this study are the MDA-MB-231 and MDA-MB-468 both of which correspond to the Triple Negative Breast Cancer (TNBC) [8,9]. These were the selection of cell lines due to the fact that they are acknowledged to express the hypoxia marker Carbonic Anhydrase IX (CA IX) receptor at the surface of the cell lines. The MDA-MB-468 is found to have a higher expression of the receptor in comparison to the MDA-MB-231 according to literature. The MDA-MB-231 is also observed to be a more aggressive cell line that presents a greater problem for treatment. MDA-MB-231 is a stellate shaped cell and the MDA-MB-468 is a grape-like cluster of cells. Both the cell lines have been cultured in Dulbecco's Modified Eagle's Medium (DMEM; Fisher Scientific, Waltham MA). The medium was mixed with 10% fetal bovine serum (FBS) and streptomycin sulfate (10 mg/L). All cell lines were incubated at 37 °C in a 5% CO_2 air humidified atmosphere.

3.7. In-vitro Cytotoxicity Studies

The in-vitro cytotoxicity studies had been executed after the cells were induced with hypoxia with the usage of $CoCl_2$. This treatment enhances the overexpression of the CA IX receptor on the surface of the cells. The assay was carried out using the MTT reagent solution in PBS (1 mg/mL) at pH 7.4. The treatments included free drug, free carrier, carrier-drug, carrier-ligand, and the system comprising carrier-ligand-drug. The cells were seeded in 96-well plates with a normal of 5000 cells in every well. After incubating these cells for 24 h, they were treated with distinctive

concentrations of the formulations within a range of 0.25 μM–5 μM. The treated cells were further incubated within the presence of the formulations for 48 h at 37 °C, and then the MTT reagent solution was added. The cells were incubated furthermore at 37 °C for 2 h. Following this, the media was supplanted by DMSO and the plates had been placed on a shaker for 10 min. The absorbance was measured at 590 nm making use of a High-Performance Multi-Mode Plate Reader (Synergy 2, BioTek). The extent of surviving cells was calculated in terms of percentage by way of contrasting the absorbance of the treated cells and proper controls cells. This was performed on each the cell lines individually. This was repeated in a hypoxic condition induced using final concentration of 100 μM $CoCl_2$ in the culture media for 24 h in the culture media. This was performed to understand the difference between the uptake of the formulation in the normoxic conditions as well as the hypoxic conditions, because healthy cells do not overexpress the CA IX receptor and do not exhibit hypoxia whilst the cancer cells do.

3.8. Drug Uptake Studies

This assay is a quantitative measure of the quantity of drug entering into the cells by the usage of fluorescence spectroscopy. Both the targeted and non-targeted formulations had been conjugated with Rhodamine B consistent with a formerly established technique from the literature. The cell lines MDA-MB-231 and MDA-MB-468 were treated with a final concentration of 100 μM $CoCl_2$ in the culture media for 24 h first, then cultured in two separate 6 well-plates with every well comprising of about 100,000 cells in 2 mL of the media. These cells were then treated with the rhodamine conjugated formulations at their respective IC50 concentrations in each cell lines and incubated. Samples were accrued every 4 h from the start of the treatment. Each sample collection procedure consisted of removing the formulation, washing the cells with PBS, and eventually streaking the cells to collect the proteins after which further protein collection was done using lysis buffer. Throughout the measurement of florescence, a control was also used that contained a mixture of methanol and the lysis buffer. These studies were also performed to study the fluorescence intensity under a fluorescence microscope.

3.9. Apoptosis Assay by Flow Cytometry

Apoptosis assay was performed on the MDA-MB-231 and MDA-MB-468 cell lines separately consistent with the previous literature. The cells had been induced with hypoxia by way of treating them with $CoCl_2$, then the cells had been cultured in 6-well plates at the rate of 100,000 cells per well. The cells had been incubated for 24 h at 37 °C under 5% CO2 condition, accompanied by using the treatment of the cells with free drug, non-targeted formulation and the targeted system and incubating further for 48 h to set off apoptosis. The concentrations of the formulations were the IC50 concentrations of the respective formulations acquired from the in-vitro cytotoxicity assay. After 48 h incubation, the cells had been collected, and the test was set up as indicated by the procedure described for Guava Nexin Annexin V assay (EMD Millipore, USA). Then, the media before trypsinization and the later treated and trypsinized cells were collected in the same 15 mL tubes for each sample and centrifuged at 800 for 5 min. Cell pellets formed consequently had been dispersed in PBS at pH 7.4 containing 1% FBS in order that the quantity of cells were of the order of 2×10^5 to 1×10^6 cells/mL. 100 μL of those cell dispersions of every sample was mixed with 100 μL of the Guava Nexin Reagent and was incubated for 20 min at room temperature in the absence of sunlight. The resultant final samples were analyzed by Guava Easycyte flow Cytometer (EMD Millipore, USA).

3.10. . In vivo TNBC PDX Tumor Targeting Efficacy in Mice

6-8 weeks old female tumor-bearing Nod/SCID mice were purchased from Jackson Laboratories, housed in a sterile environment on a standard 12-hour light−dark cycle and maintained on normal rodent diet and water. All animal procedures were approved by the Wayne State Animal Care and IACUC committee in accordance with National Institutes of Health guidelines. Six-week-old female Nod/SCID mice were injected subcutaneously with PDX of TNBC cells in PBS medium (5.0×10^6 cells per mouse). Tumor growth was measured in two perpendicular directions every 2 days with a caliper. Tumor volumes were calculated using the formula $0.5 \times a \times b^2$, where a is the measurement of the longest axis and b is the measurement of the axis perpendicular to L. Tumor bearing mice were treated via tail vein injection with 10 nmole/mice doses of BSA-DRUG-ATZS0456 and BSA-DRUG-S0456 nanoparticles, respectively. Mice were imaged 24 hours post-injection using a Carestream In vivo Extreme, Light Source: 400 W Xenon, Monochrome interlined, fixed lens (10x), cooled (-29 C, absolute), CCD camera (13.8 x 13.8 cm / 2048 x 2048 px, 67 µmpx, 16 bit), excitation 760 nm, emission 790 nm wavelength for fluorescence, and X-ray images were captured. Both fluorescence and X-ray images of mouse was merged to demonstrate the localization of nanoparticles.

REFERENCES

1. Luong, D.; Kesharwani, P.; Alsaab, H.O.; Sau, S.; Padhye, S.; Sarkar, F.H.; Iyer, A.K. Folic acid conjugated polymeric micelles loaded with a curcumin difluorinated analog for targeting cervical and ovarian cancers. *Colloids Surfaces B Biointerfaces* **2017**, *157*, 490–502.

2. Kesharwani, P.; Banerjee, S.; Padhye, S.; Sarkar, F.H.; Iyer, A.K. Hyaluronic Acid Engineered Nanomicelles Loaded with 3,4-Difluorobenzylidene Curcumin for Targeted Killing of CD44+ Stem-Like Pancreatic Cancer Cells. *Biomacromolecules* **2015**, *16*, 3042–3053.

3. Kesharwani, P.; Banerjee, S.; Padhye, S.; Sarkar, F.H.; Iyer, A.K. Parenterally administrable nano-micelles of 3,4-difluorobenzylidene curcumin for treating pancreatic cancer. *Colloids Surfaces B Biointerfaces* **2015**, *132*, 138–145.

4. Alsaab, H.; Alzhrani, R.M.; Kesharwani, P.; Sau, S.; Boddu, S.H.; Iyer, A.K. Folate decorated nanomicelles loaded with a potent curcumin analogue for targeting retinoblastoma. *Pharmaceutics* **2017**, *9*.

5. Gawde, K.A.; Sau, S.; Tatiparti, K.; Kashaw, S.K.; Mehrmohammadi, M.; Azmi, A.S.; Iyer, A.K. Paclitaxel and di-fluorinated curcumin loaded in albumin nanoparticles for targeted synergistic combination therapy of ovarian and cervical cancers. *Colloids Surfaces B Biointerfaces* **2018**, *167*, 8–19.

6. Elzoghby, A.O.; Samy, W.M.; Elgindy, N.A. Albumin-based nanoparticles as potential controlled release drug delivery systems. *J. Control. Release* **2012**, *157*, 168–182.

7. Duy Luong, Samaresh Sau, Prashant Kesharwani, A.K.I. Polyvalent Folate-Dendrimer-Coated Iron Oxide Theranostic Nanoparticles for Simultaneous Magnetic Resonance Imaging and Precise Cancer Cell Targeting. *Biomacromolecules* **2017**, *18*, 1197–1209.

8. Lock, F.E.; McDonald, P.C.; Lou, Y.; Serrano, I.; Chafe, S.C.; Ostlund, C.; Aparicio, S.; Winum, J.Y.; Supuran, C.T.; Dedhar, S. Targeting carbonic anhydrase IX depletes breast cancer stem cells within the hypoxic niche. *Oncogene* **2013**, *32*, 5210–5219.

9. Ivanova, L.; Zandberga, E.; Silin¸a, K.; Kalnin¸a, Z.; A¯bols, A.; Endzelin¸š, E.; Vendina, I.; Romanchikova, N.; Hegmane, A.; Trapencieris, P.; et al. Prognostic relevance of carbonic anhydrase IX expression is distinct in various subtypes of breast cancer and its silencing suppresses self-renewal capacity of breast cancer cells. *Cancer Chemother. Pharmacol.* **2015**, *75*, 235–246.
